# *CFAP300* mutation causing primary ciliary dyskinesia in Finland

**DOI:** 10.3389/fgene.2022.985227

**Published:** 2022-09-30

**Authors:** Rüdiger Schultz, Varpu Elenius, Mahmoud R. Fassad, Grace Freke, Andrew Rogers, Amelia Shoemark, Tiina Koistinen, Mai A. Mohamed, Jacqueline S. Y. Lim, Hannah M. Mitchison, Anu I. Sironen

**Affiliations:** ^1^ Allergy Centre, Tampere University Hospital, Tampere, Finland; ^2^ Department of Pediatrics, Turku University Hospital, University of Turku, Turku, Finland; ^3^ Great Ormond Street Institute of Child Health, University College London, London, United Kingdom; ^4^ Human Genetics Department, Medical Research Institute, Alexandria University, Alexandria, Egypt; ^5^ PCD Diagnostic Team and Department of Paediatric Respiratory Medicine, Royal Brompton Hospita, London, United Kingdom; ^6^ School of Medicine, University of Dundee, Dundee, United Kingdom; ^7^ Department of Otorhinolaryngology, Head and Neck Surgery, Kuopio University Hospital and University of Eastern Finland, Kuopio, Finland; ^8^ Biochemistry Division, Chemistry Department, Faculty of Science, Zagazig University, Zagazig, Egypt; ^9^ Natural Resources Institute Finland (Luke), Helsinki, Finland

**Keywords:** motile cilia, primary ciliary dyskinesia, CFAP300, dynein arm preassembly, diagnostics

## Abstract

Primary ciliary dyskinesia (PCD) is a rare genetic condition characterized by chronic respiratory tract infections and in some cases laterality defects and infertility. The symptoms of PCD are caused by malfunction of motile cilia, hair-like organelles protruding out of the cell that are responsible for removal of mucus from the airways and organizing internal organ positioning during embryonic development. PCD is caused by mutations in genes coding for structural or assembly proteins in motile cilia. Thus far mutations in over 50 genes have been identified and these variants explain around 70% of all known cases. Population specific genetics underlying PCD has been reported, thus highlighting the importance of characterizing gene variants in different populations for development of gene-based diagnostics. In this study, we identified a recurrent loss-of-function mutation c.198_200delinsCC in *CFAP300* causing lack of the protein product. PCD patients homozygous for the identified *CFAP300* mutation have immotile airway epithelial cilia associated with missing dynein arms in their ciliary axonemes. Furthermore, using super resolution microscopy we demonstrate that CFAP300 is transported along cilia in normal human airway epithelial cells suggesting a role for CFAP300 in dynein complex transport in addition to preassembly in the cytoplasm. Our results highlight the importance of CFAP300 in dynein arm assembly and improve diagnostics of PCD in Finland.

## Introduction

Primary ciliary dyskinesia (PCD) is an inherited, genetically and clinically heterogenous disorder caused by mutations in genes encoding proteins crucial for cilia motility. Motile cilia line the respiratory tract and are required for efficient mucus removal from the airways. Children with PCD suffer from early onset chronic airway infections and congestion leading to a distinctive, chronic wet cough (Lucas et al., 2020; Shoemark and Harman, 2021). If untreated, the condition may progress towards bronchiectasis in later life. During embryonic development, motile cilia determine the left-right positioning of body organs and nearly half of PCD patients have laterality problems. The brain ventricles are also lined with motile ependymal cilia, which are required for the flow of cerebrospinal fluid. However, hydrocephalus caused by defective ependymal cilia is rare in PCD patients. Motile cilia of the airways have a 9 + 2 axonemal core structure with a central pair of microtubules surrounded by nine peripheral doublet microtubules, which have outer and inner dynein arms (ODA and IDA, respectively) attached along their length. These ODA and IDA are motors that power cilia motility and are preassembled in the cytoplasm then transported via intraflagellar transport (IFT) to the cilium (Horani et al., 2018). Mutations in many preassembly factors have been identified as a cause for PCD in addition to mutations in structural genes of cilia components. Dynein arm preassembly factors DNAAF1 (Duquesnoy et al., 2009; Loges et al., 2009), DNAAF2 (Omran et al., 2008), DNAAF3 (Mitchison et al., 2012), DNAAF4 (Tarkar et al., 2013), DNAAF5 (Horani et al., 2012; Diggle et al., 2014), DNAAF11 (Kott et al., 2012; Horani et al., 2013; Inaba et al., 2016) and DNAAF6 (Olcese et al., 2017; Paff et al., 2017) are strictly localized in the cytoplasm, but interestingly ZMYND10 (Moore et al., 2013; Zariwala et al., 2013), CFAP298 (Austin-Tse et al., 2013; Jaffe et al., 2016) and CFAP300 (Fassad et al., 2018) are also found in the ciliary compartment, suggesting a combined role in assembly and transport. The core structure of the sperm flagellum is very similar to that of motile cilia, although some differences exist. Furthermore, male specific motile cilia are present in the efferent duct of the male reproductive tract and female specific motile cilia in the oviduct. Therefore, PCD is also associated with infertility, especially in men (Sironen et al., 2019), However, genotype/phenotype correlation between PCD and fertility is poorly understood and requires further studies.

PCD is inherited as an autosomal-recessive or rarely as X-linked disease. Thus far mutations in over 50 genes have been identified to cause PCD, explaining approximately 70% of cases. Over 300 genes encode essential components of the motile ciliary machinery and a large number of patients are still without a genetic diagnosis (Lucas et al., 2020). The aim of this study was to identify disease causing variants in the Finnish PCD population, where until now only two variants in *DNAH11* have been previously reported (Schultz et al., 2020). It is important to increase the understanding of genetic causes for PCD, which can be most effectively traced in a genetically isolated population such as the Finns. This study contributes to the understanding of genetic causes of PCD by identification of a causative loss of function (LoF) mutation in *CFAP300* in Finnish PCD patients. Population specific mutations play an important role in PCD and thus it is important to investigate the genetic variability in different populations (Fassad et al., 2020; Mitchison and Smedley, 2022). We also show that additional to a role in the cytoplasmic preassembly of dynein arms, human CFAP300 is involved in protein transport to motile cilia, which was previously only suggested in model organisms.

## Methods

### Subject

Blood samples were collected from thirteen PCD patients, recruited at the University Hospitals of Turku, Kuopio and Tampere after written informed consent was given. For variant segregation analysis saliva samples were collected from families (healthy parents and siblings when possible). Patients were interviewed and scored using the PICADAR questionnaire (Behan et al., 2016). The study was ethically approved by the University of Turku Ethics Committee (ETMK 69–2017), London Bloomsbury Research Ethics Committee approved by the Health Research Authority (08/H0713/82), and the referring hospitals.

### Nasal nitric oxide analysis

For nasal nitric oxide (nNO) analysis a CLD 88sp analyser equipped with a Denox 88 module for flow control was used (Eco Physics, Dürnten, Switzerland). If cooperativity was established, three consecutive trials were taken, from which the highest value was recorded. Nasal nitric oxide analysis was repeated on two different occasions.

### High-speed video microscopy analysis

Nasal epithelial cells were suspended in DMEM medium and evaluated under a differential-interference microscope (Zeiss, Oberkochen, Germany) at ×1,000 magnification and cilia beat was recorded with a digital high-speed video (DHSV) camera (Hamamatsu Orca Flash 4.0, Hamamatsu Japan) with a frame rate of 256 Hz. The detailed protocol for HSVM can be found in Schultz et al., 2022. DHSV video sequences were played back frame by frame and cilia beat frequency (CBF) was determined by calculating the mean of all recorded cilia beat cycles and the cilia beating pattern (CBP) was determined by two independent expert operators. HSVM was repeated on two different occasions.

### Whole exome sequencing

The Nonacus Cell3 Exome panel was used for whole exome sequencing of patient samples (https://nonacus.com/cell3tm-target ). Unmapped reads were aligned to the current Human reference genome (GRCh38 build) by Burrows-Wheeler Aligner tool (Bwa-mem2 version) (Li and Durbin, 2010), SAM files were produced and indexed and converted to BAM format previous to marking and removing duplicates using Picard (https://broadinstitute.github.io/picard/). Subsequent analysis was executed following best practices guidelines for GATK from the Broad Institute (https://gatk.broadinstitute.org/hc/en-us). Firstly, base quality score recalibration (BQSR) was done to numerically correct individual base calls. Variant discovery was carried out in a two-step pipeline: variant calling with HaplotypeCaller followed by joint genotyping with GenotypeGVCFs. Once obtained a multi-sample VCF file containing all definitive variant records, VariantRecalibrator was operated to fulfil Variant Quality Score Recalibration (VQSR) and refinement of the obtained variant callset.

Variants were individually selected for each sample. The called variants, including both single nucleotide variants (SNVs) and indels, were then annotated using ANNOVAR (http://www.openbioinformatics.org/annovar/). Such tool enables functional annotation, and thus obtaining final VCF files that contain detailed information for each variant site in the sample, such as their impact within a gene, predicted pathogenicity scores, minor allele frequency (MAF), zygosity status or reporting whether they have been recorded in large-scale databases like dbSNP.

### Variant prioritization

Variants were filtered for MAF <1%, their predicted functional impact on the encoded protein (missense, splicing, frameshift or nonsense) and frequency (<0.001) in the Genome Aggregation Database (gnomAD, broadinstitute.org). A list of genes with high expression during motile cilia development (*n* = 652, reanalyzed gene list based on data in Marcet et al., 2011) was used to further filter variants in genes with potential roles in motile cilia. Finally, the pathogenicity of the identified variants was estimated with Combined Annotation Dependent Depletion tool (CADD, https://cadd.gs.washington.edu/snv) and a CADD score >20 was considered significant pathogenicity score. Due to the known inheritance pattern of PCD homozygous variants were prioritized, but output files were also analyzed for the presence of compound heterozygous variants.

### Sanger sequencing

The identified NM_032930.3 *CFAP300* variant was confirmed in the probands and segregation within family members by Sanger sequencing. Primers flanking the mutation were designed using the NCBI Primer-BLAST tool, forward GTA​TGT​CAG​TTG​TTA​CGA​AGG​CAA​T, reverse TGC​TCT​TAT​GTG​TTA​AGC​CAG​C. The genomic sequence was amplified using standard PCR conditions and predicted primer annealing temperature. The specificity of the PCR product was confirmed on agarose gel and purified using Exosap for Sanger sequencing.

### Electron microscopy

Samples were fixed in 2.5% glutaraldehyde in cacodylate buffer and processed for electron microscopy. Defects were quantified using the method described by Shoemark et al. (2012). Briefly, cells were washed in sodium cacodylate buffer, post-fixed with 1% osmium tetroxide and centrifuged in agar or agarose to generate a pellet. Using a series of increasing concentrations of methanol followed by propylene oxide, cells were dehydrated before embedding in resin then 70–90 nm sections were cut using an ultramicrotome, mounted onto copper grids. Heavy metal staining was performed with uranyl acetate and lead citrate. Assessment of the respiratory epithelium and ciliary ultrastructure was made on a Jeol 1500 transmission electron microscope (TEM). Quantification of cells, microtubular arrangement in the axoneme and the presence of dynein arms was performed by a clinical electron microscopist blinded to the case information. Care was taken to assess cilia from a number of healthy cells from locations proximal and distal to the epithelial cell surface. Transverse sections of cilia were methodically quantified until either the entire section or >200 cilia had been counted.

### Immunofluoresence

Nasal ciliated cells from nasal brushing of a PCD patient with *CFAP300* mutations and a control sample were stained after blocking (10% BSA, in PBS) using DNAH5 (HPA037470, Cambridge Bioscience), DNAH7 (HPA037724, Atlas Antibodies), DNAI1 (HPA021649, Atlas Antibodies), RSPH4A (HPA031196, Sigma-Aldrich) and CFAP300 (c11orf70, HPA038585, Sigma-Aldrich) antibodies and colocalized with cilia marker alpha-tubulin (322588, Invitrogen). After washes with PBST the slides were incubated with secondary antibodies Alexa Fluor 488 anti-mouse and Alexa Fluor 594 anti-rabbit (1:500). Slides were imaged using Zeiss Axio Observer seven and deconvoluted using Huygens Deconvolution software (https://svi.nl/Huygens-Deconvolution). High resolution images were taken using a Zeiss LSM 880 upright confocal multiphoton microscope with AiryscanFast using ×63 objective and excitation lasers for 488 and 594. Images were processed using Zen software.

### Statistical analysis

Unpaired *t*-test was used for statistical analysis between patient and control samples. *p*-value < 0.05 was considered statistically significant.

## Results

### Clinical identification of PCD patients

Patients were diagnosed based on low nasal NO, altered ciliary beating pattern in HSVM and clinical patient data (Table 1). Cystic fibrosis was excluded for all patients using a sweat test. All patients showed classic PCD symptoms early in life including chronic wet cough and otitis media, but to date no bronchiectasis has been observed (Table 1). Pneumonia was detected in one patient later in life, who had a low Picadar score (4/12, patient III-1). The low Picadar score was probably due to lack of situs defects in this patient. Defective beating of airway cilia was observed by HSVM showing stiff or static cilia in all patients. Some residual unsyncronized movement was detected in patient III-1. This variation in ciliary beating might be due to variable levels of dynein arms, however no samples for electron microscopy or immunofluorescence were available from patient III-1 preventing more detailed examination.

**TABLE 1:** PCD patient characteristics for patients with LoF mutations in *CFAP300*.

Patient ID	Year of birth	Sex	Cilia beating frequency (Hz)	Beating pattern in HSVM	Nasal NO	Situs inversus	Picadar score	Clinical symptoms	TEM
I-1	2014	F	0	static with weak residual movement	8	Yes	na	Chronic wet cough, otitis media	na
II-1	2016	M	0	stiff, nearly static	na	Yes	10/12	Chronic wet cough, otitis media	lack of ODA and IDA
III-1	1998	F	12.2	stiff, unsynchronized	21	No	4/12	Cronic wet cough, recurrent otitis media and pneumonia	na

na: not available.

### Whole exome sequencing identified patients carrying variants in *CFAP300*

**FIGURE 1:**
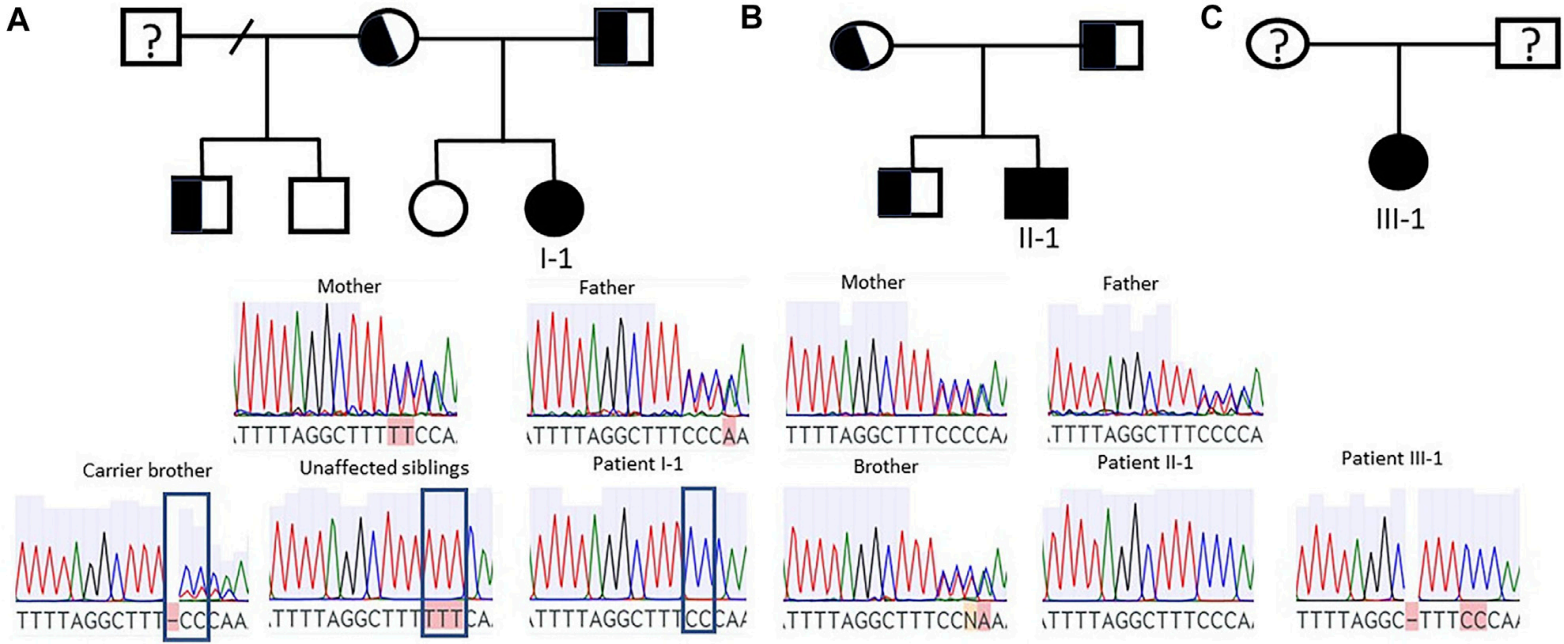
Segregation of *CFAP300* mutations. Patients I-1 and II-1 were homozygous for the mutation while parents were heterozygous. **(A)**. Patient I-1 family with carrier parents and one carrier brother and two unaffected siblings. The mutation site is indicated in carrier and unaffected siblings and in patient I-1. **(B)**. Patient II-1 family with carrier parents and brother. **(C)**. Sanger sequencing result for patient III-1.

**FIGURE 2:**
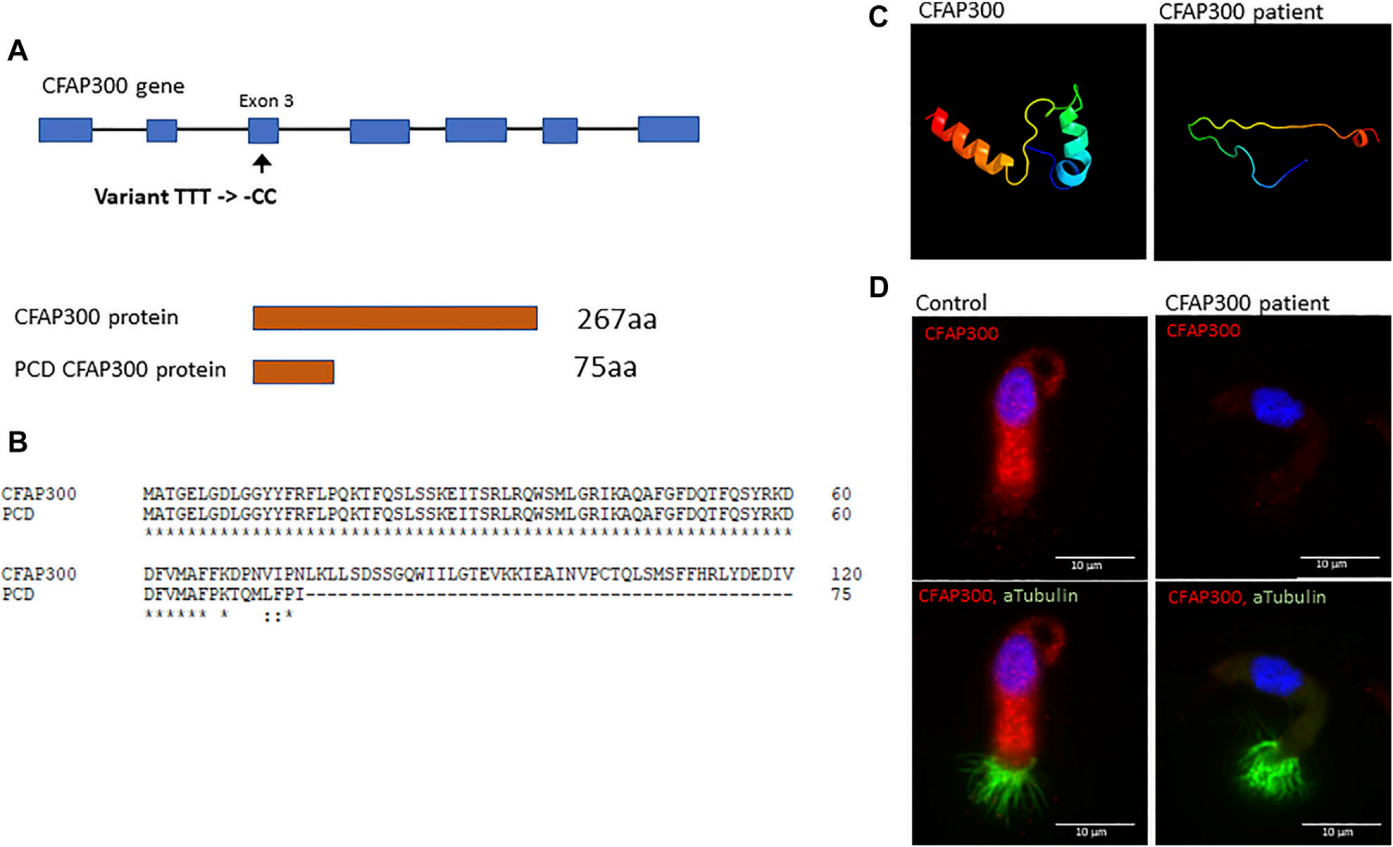
The effect of the *CFAP300* variant on protein function. **(A)**. The *CFAP300* variant **(C)**.198_200delinsCC results in premature stop codon at 76 aa and protein product. **(B)**. Clustal Omega alignment of the control and PCD patient CFAP300 protein sequence. **(C)**. Structural changes in the CFAP300 protein folding based on Phyre2. **(D)**. Cytoplasmic localization of the CFAP300 protein product appears missing in the PCD patient (patient II-1). αTubulin was used as a marker for cilia and Dapi as a nuclear stain. Scale bar is 10 µm.

We conducted whole exome sequencing of 13 Finnish PCD patients, which identified a homozygous LoF mutation in exon 3 of the *CFAP300* gene. The variant was present in homozygous state in three unrelated Finnish patients with typical symptoms of PCD including chronic respiratory infections and static/stiff cilia in HSVM and two of the patients had *situs inversus* (Table 1). No other candidate homozygous or compound heterozygous variants were detected in these patients. The presence of the mutations was confirmed by Sanger sequencing and it also segregated consistently in two of the families where familial samples were available (Figure 1, samples unavailable for the third family). Sanger sequencing confirmed a 3bp deletion and insertion of CC at chr11:102058886-102058888, causing a frameshift c.198_200delinsCC predicted to cause a premature stop codon at position 76 in the protein sequence, p. Phe67fs (Figure 2A). The variant is not present in gnomAD and it seems to be specific to PCD patients as it has previously been reported in German, Israeli and Slavic populations (Höben et al., 2018; Zietkiewicz et al., 2019), providing strong evidence that the variant is disease causing based on American College of Medical Genetics and Genomics (ACMG) guidelines (Richards et al., 2015). Protein alignment showed the premature stop codon position after 75 aa (Figure 2B). The effect of the frameshift on protein structure was predicted by Phyre2 (PHYRE2 Protein Fold Recognition Server (ic.ac.uk)), which showed lack of helical structures in the patient protein prediction (Figure 2C). Based on the *in silico* analysis, it can be predicted that the identified frameshift mutation in *CFAP300* is disease causing.

### CFAP300 is missing in the PCD patient with a frameshift mutation resulting in a lack of dynein arms

**FIGURE 3:**
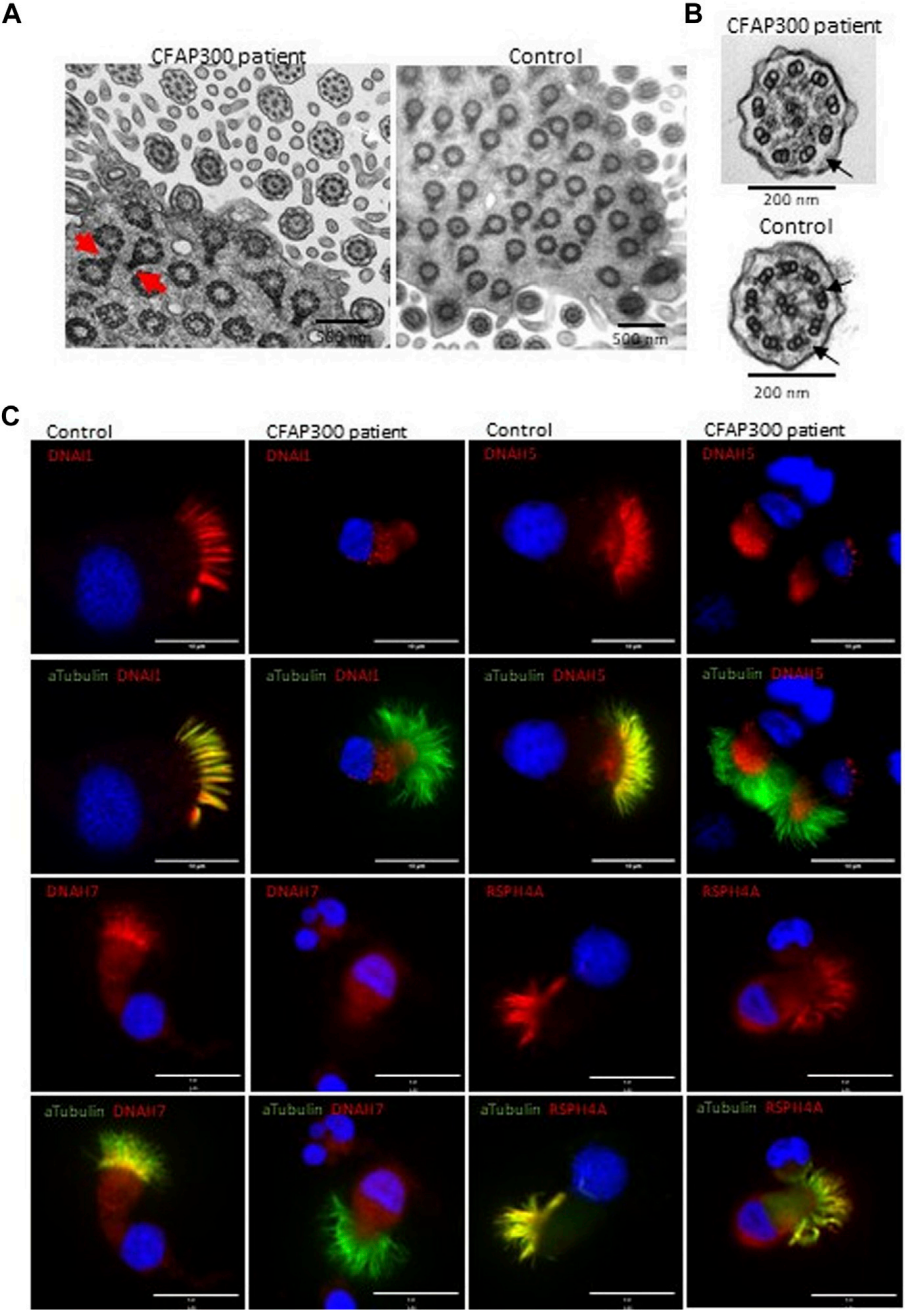
Localization of axonemal proteins in airway cilia of a control and PCD patient (patient II-1) with *CFAP300* mutations. **(A)**. High number of cilia cross sections were present in the nasal brushing sample of a PCD patient with *CFAP300* c.198_200delinsCC mutations, but the orientation of the basal foot (red arrows) was disorganized compared to control. **(B)**. The main finding in TEM of cilia cross sections in a patient with the *CFAP300* variant was lack of dynein arms (black arrows). **(C)**. ODA (DNAH5 and DNAI1) and IDA (DNAH7) proteins were missing from cilia in the patient airway epithelial cilia, while strong staining is present in the control sample. Radial spoke protein RSPH4A is present in the patient cilia. αTubulin was used as a marker for cilia and Dapi as a nuclear stain. Scale bar is 10 µm.

To confirm the functional effect of the identified frameshift variant in *CFAP300*, we investigated the localization of the protein product in control and patient ciliated airway epithelial cells. In control ciliated airway epithelial cells CFAP300 localized in the cytoplasm, which is consistent with its predicted function as a dynein arm preassembly factor (Figure 2D). CFAP300 staining was not detected or extremely weak in cells from patients homozygous for the c.198_200delinsCC variant compared to the control, consistent with likely nonsense mediated decay of the defective mRNA transcripts (Figure 2D). The peptide sequence for CFAP300 antibody corresponds to 113-188 aa in the CFAP300 protein sequence (ENSP00000414390), which is expected to be affected by the predicted premature stop codon at position 75 aa. The weak staining detected in patient cells could potentially originate from a short protein isoform of CFAP300 containing 109-262 aa (ENST00000529204). TEM analysis showed a high number of ciliated cells and normal arrangement of microtubules in the PCD patient with *CFAP300* LoF mutations. However, the orientations of the basal feet were disorganized (Figure 3A). For a synchronized beating pattern the basal feet at the motile cilia base all have to be organized to the same directions in all cilia (Nguyen et al., 2020). This defect may be secondary, due to the lack of cilia motility in the patient and additional studies are needed to confirm the order of events of basal foot orientation in the presence of the *CFAP300* frameshift variant. The main structural change in cilia cross sections was the lack of dynein arms (Figure 3B). The cross-sectional morphology of cilia appeared abnormal with a partial to total absence of ODA and IDA. Both dynein arms were occasionally present as complete structures or truncated ‘horns’ in the case of ODA. Ciliary cross sections with majority of arms absent were counted as both arms absent. In the patient with *CFAP300* mutation no cross sections with normal dynein arms were detected, which was statistically significant compared to control (>97% cross sections with normal dynein arms). No significant microtubular disorganization was detected in TEM. The lack of dynein arms was confirmed by immunofluorescence using antibodies against IDA protein DNAH7 and ODA proteins DNAI1 and DNAH5. These proteins appeared completely missing in patient cilia with strong staining retained in the cytoplasm (Figure 3C). In the control sample, DNAH7, DNAH5 and DNAI1 were specifically localized along the cilia. Furthermore, radial spoke protein RSPH4A was present in the patient cilia, although staining was also detected in the cytoplasm (Figure 3C). This may be due to inhibited transport of proteins to the cilium in patients with the *CFAP300* mutation, but the mutational mechanism requires additional studies.

### CFAP300 is transported to cilia in airway epithelial cells

**FIGURE 4:**
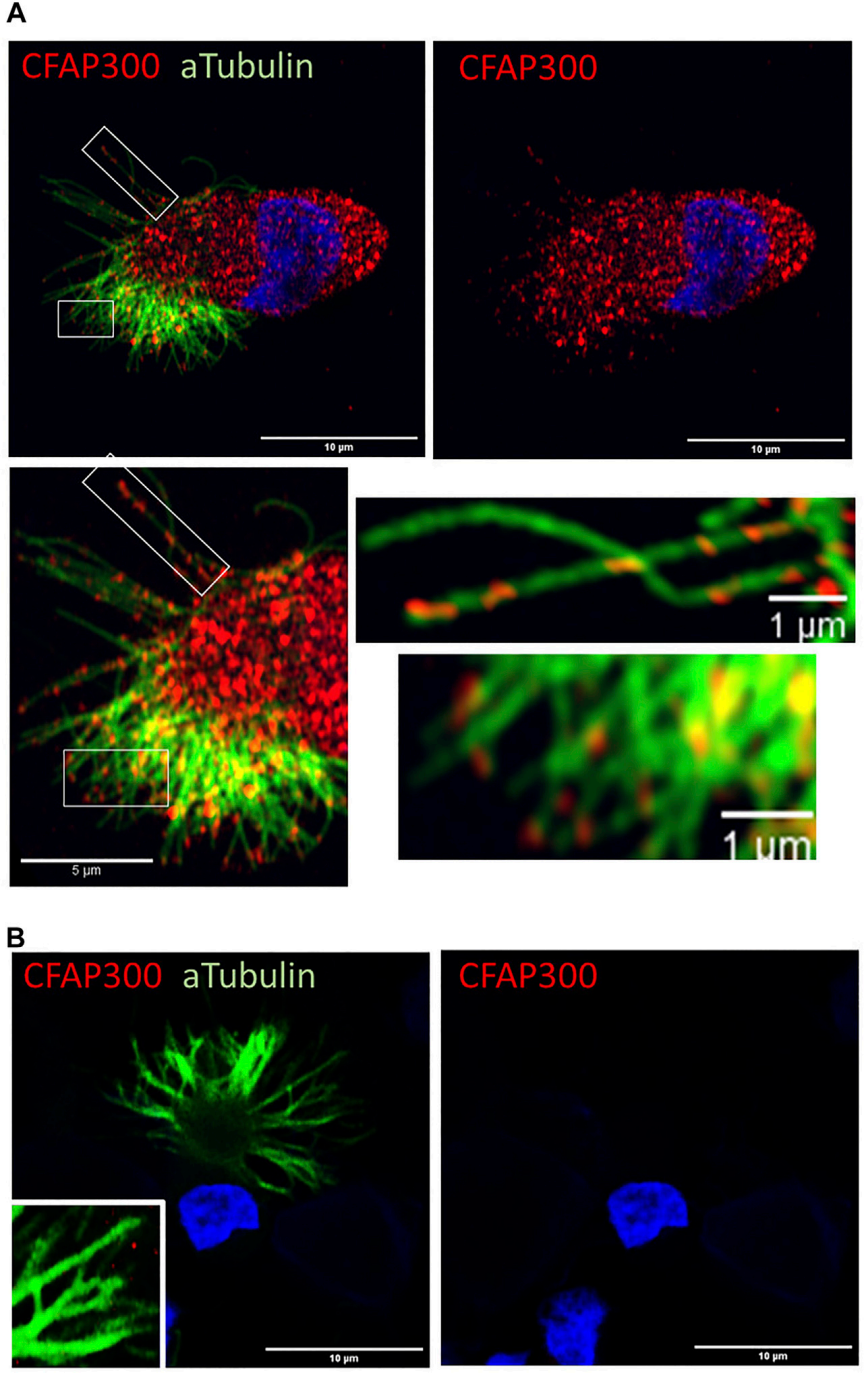
CFAP300 is transported along the motile airway epithelial cilium. **(A)**. In normal airway epithelial cells most of the CFAP300 protein is localized in the cytoplasm, but some of the protein appears to be transported into the cilium. **(B)**. No CFAP300 staining was detected in the patient airway epithelial cells.

Using Airyscan super resolution imaging we characterized the strong cytoplasmic staining of CFAP300 across the cytoplasm, which was present as granular spots in control ciliated airway epithelial cells. Previously, it has been shown in model species (*Paramecium* and *Chlamydomonas*) that a small amount of CFAP300 is also transported to the cilium (Fassad et al., 2018). To investigate the potential localization of CFAP300 in human cilia in addition to its strong staining in the cytoplasm (Figure 2D), we focused on imaging of CFAP300 along the cilium. We found that CFAP300 was present along the ciliary axoneme indicating that it is also transported to the cilium (Figure 4). Both cytoplasmic and cilia CFAP300 staining was depleted in the PCD patient sample with LoF mutations in *CFAP300* (Figure 2D).

## Discussion

In this study we have identified a mutation in *CFAP300* causative for PCD in Finland and analysed the effect of the mutation on motile cilia formation and function. The main defect in axonemal cross-sections was the lack of dynein arms as described in previous studies (Fassad et al., 2018; Höben et al., 2018; Zietkiewicz et al., 2019; Aprea et al., 2021; Bolkier et al., 2021; Yiallouros et al., 2021). Furthermore, we detected misorientation of the basal feet of the airway cilia, which we believe is most likely to be a secondary defect due to the lack of cilia motility and is also seen in PCD cases due to mutations in other genes. Previously it has been shown that cilia motility is required for correct polarization of basal bodies (Mitchell et al., 2007). However, it cannot be ruled out that CFAP300 has a more direct role in basal body polarization, as has been suggested for another dynein arm preassembly factor CFAP298 (Jaffe et al., 2016). We have demonstrated the main localization of CFAP300 to be in the cytoplasm of normal ciliated airway epithelial cells, but we find in addition the presence of small amount of CFAP300 along the ciliary length (Figure 4) suggesting that CFAP300 has a potential role in transport of dynein complexes into the cilium. These results are consistent with previous studies in model organisms Paramecium and *Chlamydomonas*, where CFAP300 orthologs have been shown to be mainly present in the cytoplasm with small amount along the cilia (Fassad et al., 2018). Gene silencing experiments indicated that localization of CFAP300 along the axoneme is IFT dependent (Fassad et al., 2018). These studies suggest that CFAP300 is involved in chaperone-mediated preassembly of dynein arms and transporting these complexes to motile cilia, which is supported by our results in human cells.

Previously children with *CFAP300* mutations have been shown to share a consistent PCD phenotype from early life with laterality defects and immotile respiratory cilia displaying combined loss of IDA and ODA (Fassad et al., 2018). Finnish PCD patients with *CFAP300* LoF mutations showed a similar phenotype with laterality defects in two of the patients and consistent chronic wet cough. The respiratory distress was already evident in the neonatal care unit and HSVM showed static or stiff cilia with loss of dynein arms as described previously. The variant identified in this study has also been previously reported in one German and Israeli case (Höben et al., 2018) and 14 PCD patients in the Slavic population (Zietkiewicz et al., 2019), which along with this study indicates that it is a reasonably common cause of CFAP300 PCD that could arise from an ancient European founder mutation. ODA loss was reported in 67–95% and IDA loss in 75–95% of axonemal cross sections in patients with homozygous missense mutations at c.776A>G (p.His259Arg) and compound heterozygous stop gain mutations at c.154C>T and c.361C>T (p.Gln52*, p. Arg121*), respectively (Fassad et al., 2018). DNAH5 and DNALI1 staining was missing in patient cilia, but RSPH4A and GAS8 staining was comparable to controls (Fassad et al., 2018). In a patient with a homozygous stop gain mutation c.361C>T (p.Arg121*) in *CFAP300*, DNAI1, DNAI2 and DNALI1 were reported to be missing in cilia and TEM reported only ODA defect (Aprea et al., 2021). In frame deletion c.98_106del (p.Arg33_Arg35del) in six patients of Greek-Cypriot population was associated with IDA and ODA loss in most cilia cross-sections based on TEM and there was an extremely stiff CBP (Yiallouros et al., 2021). The c.198_200delinsCC frameshift variant identified in this study caused lack or truncation of IDA or ODA in all axonemal cross sections based on TEM of patient II-1 and lack of DNAH5, DNAI1 and DNAH7 suggesting a severe defect in dynein arm assembly. Patient III-1 showed stiff cilia movement with a beat frequency of 12 Hz. Although we were unable to confirm the axonemal defects in this patient, the residual movement could be due to different levels of dynein arm formation as has been shown in previous studies (Fassad et al., 2018; Aprea et al., 2021; Yiallouros et al., 2021). Variable cilia beat frequency has also been reported for variant c.98_106del (0–9.1 Hz, Yiallouros et al., 2021). All the *CFAP300* mutations reported to date are listed in Table 2.

**TABLE 2:** *CFAP300* mutations identified in PCD patients.

Population	Variant	Protein	Zygosity	Number of patients	Cilia motility	Functionality	References
Israel	c.198_200 delinsCC	p.Phe67Profs*10	Homozygous	1	na	Frameshift > stop gain	Höben et al. (2018)
Germany	c.198_200 delinsCC, c.433A>T	p.Phe67Profs*10, p.Arg145*	Homozygous	2	immotile/na	Stop gain	Höben et al. (2018)
Turkey	c.361C>T	p.Arg121*	Homozygous	1	immotile	Stop gain	Höben et al. (2018)
Italy	c.361C>T	p.Arg121*	Homozygous	1	immotile	Stop gain	Höben et al. (2018)
Pakistan	c.776A>G	p.His259Arg	Homozygous	2	immotile	Missense	Fassad et al. (2018)
India	c.154C>T, c.361C>T	p.Gln52∗, p.Arg121∗	Compound heterozygous	1	immotile	Stop gain	Fassad et al. (2018)
not known	c.361C>T	p.Arg121*	Homozygous	1	immotile	Stop gain	Aprea et al. (2021)
Poland	c.198_200 delinsCC	p.Phe67Profs*10	Homozygous	14	na	Frameshift > stop gain	Zietkiewicz et al. (2019)
Poland	c.198_200 delinsCC, c.353A > G	p.Phe67Profs*10, p.Asp118Gly	Compound heterozygous	1	na	Frameshift > stop gain, exonic splice site	Zietkiewicz et al. (2019)
Poland	c.198_200 delinsCC, c.675 + 3_6 delAAGT	p.Phe67Profs*10, na	Compound heterozygous	1	na	Frameshift > stop gain, intronic splice site	Zietkiewicz et al. (2019)
Greece-Cyprus	c.95_103delGCCGGCTCC	p.Arg33_Arg35del	Homozygous	6	immotile, stiff	Inframe deletion	Yiallouros et al. (2021)
Arab–Muslim	c.693_694insGA	p.Cys231fs	Homozygous	2	na	Frameshift	Bolkier et al. (2021)
Finland	c.198_200 delinsCC	p.Phe67Profs*10	Homozygous	3	immotile, stiff	Frameshift > stop gain	This study

In the Finnish population two novel mutations in *DNAH11* have been previously reported, suggesting a distinct genetic pool for PCD in Finland. The *CFAP300* mutation reported here in contrast appears to be common with Slavic, German and Israeli populations, suggesting a common genetic origin. It is important to establish the genetic causes of PCD in different populations including Finland to improve the genetic diagnosis of PCD. This can lead to development of a population specific gene panel for the diagnostic pipeline and the opportunity for targeted specific personalised medicines in future.

Our results and previously reported studies in model organisms and PCD patients underline the crucial role of CFAP300 in dynein arm preassembly and transport to motile cilia. The exact role of CFAP300 requires additional studies in mammalian species to establish the molecular mechanisms behind the preassembly and transport of dynein arm complexes. Interestingly, CFAP300 seems to be involved in transporting the complexes along the cilia possibly acting as a linker between the IFT machinery and dynein motor complexes.

## Data Availability

The data presented in the study are deposited in the UCL Research Data Repository DOI 10.5522/04/20793235.
